# *Fukuyoa paulensis* gen. et sp. nov., a New Genus for the Globular Species of the Dinoflagellate *Gambierdiscus* (Dinophyceae)

**DOI:** 10.1371/journal.pone.0119676

**Published:** 2015-04-01

**Authors:** Fernando Gómez, Dajun Qiu, Rubens M. Lopes, Senjie Lin

**Affiliations:** 1 Laboratory of Plankton Systems, Oceanographic Institute, University of São Paulo, São Paulo, Brazil; 2 CAS Key Laboratory of Tropical Marine Bio-resources and Ecology, South China Sea Institute of Oceanology, Chinese Academy of Science, Guangzhou, China; 3 State Key Laboratory of Marine Environmental Science and Marine Biodiversity and Global Change Research Center, Xiamen University, Xiamen, China; 4 Department of Marine Sciences, University of Connecticut, Groton, Connecticut, United States of America; University of New South Wales, AUSTRALIA

## Abstract

The marine epiphytic dinoflagellate *Gambierdiscus* is a toxicologically important genus responsible for ciguatera fish poisoning, the principal cause of non-bacterial illness associated with fish consumption. The genus currently contains species exhibiting either globular or anterior-posteriorly compressed morphologies with marked differences in cell shape and plate arrangement. Here we report a third globular, epiphytic and tychoplanktonic species from the coasts of Ubatuba, Brazil. The new species can be distinguished from *G*. *yasumotoi* and *G*. *ruetzleri* by its broader first apical plate that occupies a larger portion of the epitheca. Accordingly, phylogenetic trees from small subunit (SSU) and large subunit (LSU) ribosomal DNA sequences also showed strongly supported separation of the new species from the *G*. *yasumotoi / G*. *ruetzleri* group albeit with short distance. The molecular phylogenies, which included new sequences of the planktonic species *Goniodoma polyedricum*, further indicated that the globular species of *Gambierdiscus* formed a tight clade, clearly separated (with strong bootstrap support) from the clade of lenticular species including the type for *Gambierdiscus*. The morphological and molecular data in concert support the split of *Gambierdiscus sensu lato* into two genera. *Gambierdiscus sensu stricto* should be reserved for the species with lenticular shapes, highly compressed anterioposteriorly, with short-shank fishhook apical pore plate, large 2' plate, low and ascending cingular displacement, and pouch-like sulcal morphology. The new genus name *Fukuyoa* gen. nov. should be applied to the globular species, slightly laterally compressed, with long-shank fishhook apical pore plate, large 1' plate, greater and descending cingular displacement, and not pouch-like vertically-oriented sulcal morphology. *Fukuyoa* contains the new species *Fukuyoa paulensis* gen. et sp. nov., and *F*. *yasumotoi* comb. nov. and *F*. *ruetzleri* comb. nov.

## Introduction

Ciguatera fish poisoning is the principal cause of non-bacterial illness associated with the consumption of fish contaminated with ciguatoxins. These lipophilic toxins are produced by species of the dinoflagellate genus *Gambierdiscus* and are bioaccumulated in marine food webs. Ciguatera affects approximately 25,000–500,000 people annually around the world and represents one of the most important constraints on development of tropical and sub-tropical fisheries [1,2,3,4]. Species from the genus *Gambierdiscus* may also produce other toxins such as maitotoxins, gambierol and gambieric acid [5,6]. Knowledge of the taxonomy, distribution and ecology of *Gambierdiscus* is essential to address this human health issue and improve our understanding of what causes ciguatera fish poisoning events.

The most extended hypothecal tabulation of dinoflagellates comprises five or six postcingular plates and two antapical plates [7]. The generic split is usually based on the epithecal plates because there is a higher variability in the plate number and arrangement. In addition to the apical pore plate, the goniodomatacean genera *Alexandrium*, *Coolia*, *Gambierdiscus* and *Goniodoma* contain ten epithecal plates. A discrepancy exists among different authors on the plate nomenclatures because the same species can be reported with three apical and seven precingular plates, or four apical and six precingular plates. The main problem concerns the first apical plate (1') which in some species (i.e. *Alexandrium*) should be called first precingular plate instead of first apical, because it does not touch the apical pore plate (see more details in Fraga et al. [6]). To date, twelve species of *Gambierdiscus* have been described. The type species, *Gambierdiscus toxicus*, and ten other species had anterioposteriorly compressed cell bodies with lenticular shapes [6,8,9,10,11,12,13]. In 1999, Holmes [14] described a new *Gambierdiscus* species (*G*. *yasumotoi*) with a distinct globular rather than anterioposteriorly compressed morphology. This species was also notably smaller in size than the previously described lenticular species, with a different cingular displacement (descending), a different shape of the apical pore plate (long-shank fishhook-shaped slit), and different arrangement of sulcal lists (non-converging; not pouch-like). A second globular species, *G*. *ruetzleri*, was further placed into *Gambierdiscus* [11]. The taxon sampling in the published molecular phylogenies of *Gambierdiscus* has been limited to the genera *Alexandrium*, *Coolia* and *Ostreopsis*, whereas sequences of other close relatives as such as *Goniodoma* are missing. These molecular phylogenies have revealed that *G*. *yasumotoi* and *G*. *ruetzleri* formed a separate clade basal to the typical lenticular species of *Gambierdiscus* [11,15]. Based on the observed early divergence of the globular species in molecular phylogenies, they have been considered evolutionary intermediates between a more ancestral globular morphotype and the lenticular forms, and perhaps members of different genera [11]. The globular morphology of *G*. *ruetzleri* and *G*. *yasumotoi* resembles *Alexandrium*, *Coolia* or *Goniodoma* more than the anterioposteriorly compressed species of *Gambierdiscus* [11].

In this first detailed taxonomical study of the genus in the South Atlantic Ocean, we propose a new species of globular *Gambierdiscus*. We compared the tabulation and phylogenetic relationship of lenticular and globular *Gambierdiscus* and *Goniodoma*. We obtained the first SSU rDNA sequence of *Goniodoma* and an additional LSU rDNA sequence. The molecular phylogeny revealed that the new species was basal to the group of globular species of *Gambierdiscus*. When the sequences of *Goniodoma* were included, the molecular phylogenies inferred from LSU rDNA grouped *Goniodoma* and the globular species in a clade, and the typical lenticular species of *Gambierdiscus* in another clade. These molecular phylogenies confirm the well-known important differences observed in the morphology between globular and lenticular species. The placement of the globular species within the genus *Gambierdiscus* is not supported, and we propose a new genus for these globular species.

## Materials and Methods

### Ethics Statement

The locations of the field studies are not privately owned or protected in any way. No activity during field study involved any endangered species or protected species.

### Sampling, isolation and culturing

Samples were collected in December 2013 during the low tide around the pier of the Marine Station of the University of São Paulo at Ubatuba, São Paulo State, Brazil (23° 30' 3.09" S, 45° 7' 7.32" W). The upper centimeter of sandy sediments or macroalgal specimens were placed into a 250 mL bottle with ambient water and stirred vigorously. The gross particles were removed through a 200-μm mesh filter. In the laboratory, the bottle sample was stirred, and the suspension was let settle in a composite settling chamber. The settled material was examined with a Nikon TS100 inverted microscope (Nikon, Tokyo, Japan) and photographed with a Sony Cyber-shot digital camera (model DSC-W300). Individual *Gambierdiscus* cells were isolated using a micropipette and placed in 24-well tissue culture plate with 0.2 μm-filtered seawater collected that day from the same locality, and supplemented with f/2 medium without silicates. Two days later, the healthy specimens were re-isolated and placed into a 6-well tissue culture plate with f/2 medium made with filtered and sterilized seawater collected two kilometers offshore, and incubated at 23°C, with 100 μmol photons m^−2^ s^−1^ from cool-white tubes; the photoperiod was 12:12 h L:D. The cultures were transferred to 50 mL polystyrene tissue culture flasks. The strain was deposited at the Culture Collection of Microalgae (CCVIEO) of the Instituto Español de Oceanografía in Vigo, Spain, under accession number VGO1185; Banco Español de Algas (BEA) Spain, under accession number BEA 1203B; and the microalgae culture collection of University of Xiamen, Xiamen, China, under accession number CCMA404. The strain will be available at Provasoli-Guillard National Center for Marine Algae and Microbiota (NCMA) at Bigelow Laboratory for Ocean Sciences, USA. The culture strain is also available at the Oceanographic Institute of the University of São Paulo, São Paulo, Brazil, on request to F. Gómez and R.M. Lopes, available at the Chinese Academy of Science, Guangzhou, China, on request to D. Qiu, and the University of Connecticut, Groton, USA, on request to S. Lin.

For molecular studies, cells of *Gambierdiscus* from the culture were micropipetted individually with a fine capillary into a clean chamber and washed several times in a series of drops of 0.2 μm-filtered and sterilized seawater. A total of 50 cells were placed in a 0.2 mL Eppendorf tube filled with several drops of absolute ethanol. The sample was kept at room temperature and in darkness until the molecular analysis could be performed. Specimens of *Goniodoma* were collected from surface waters using a phytoplankton net (20 μm mesh size) in February 2014 off Ubatuba, São Paulo State, Brazil (23° 32' 20.15" S, 45° 5' 58.94" W). The attempt to culture *Goniodoma polyedricum* was unsuccessful. For molecular studies, ten to 20 cells of *G*. *polyedricum* from the net sample were isolated using the method described above.

### Light microscopy

Light microscopy observations of live cells of *Gambierdiscus* were carried out under at ×1000 magnification with a Zeiss Imager.A2 microscope equipped Nomarski differential interference contrast (D.I.C.) optics. Cells fixed with glutaraldehyde (5% final concentration) were stained with DAPI (4',6' diamino-2-phenylindole) and observed at ×1000 magnification under an Olympus BX51 epifluorescence microscope equipped with an Olympus DP72 camera (Olympus, Tokyo, Japan). For plate pattern identification, the cells from cultures were dissected, squashed by gently pressing the cover slip over them, and occasionally with the aid of sodium hypochlorite (2%) and observed at ×1000 magnification under an Olympus BX51 microscope. Cells fixed with glutaraldehyde were stained with both SYBR Green I (green emission) and Fluorescent Brightener 28 (blue emission) and observed at ×630 magnification with an inverted Confocal Leica TCS SP8 AOBS microscope. The nomenclature for the plate tabulation followed Litaker et al. [11]. Cell size was measured by light microscopy on 15–20 randomly selected living cells. Cell size was described as depth (ventral to dorsal distance), width (transdiameter), and length (apical to antapical axis measured in either ventral or dorsal view).

### Scanning electron microscopy

To preserve the *Gambierdiscus* cells, glutaraldehyde (50% solution) was added to the culture to make a final concentration of 5%. Cells were filtered onto a 0.8 μm or 3 μm pore size Nuclepore membrane filter, washed with distilled water, fixed with osmium, dehydrated with a graded series of ethanol (30%, 50%, 70%, 90%, 95%, 99%, 100%) and critical-point-dried with CO_2_. Filters were mounted on stubs, sputter-coated with gold and viewed under a Phillips XL30 (FEI Company, Hillsboro, OR, USA) or a Zeiss Sigma FE (Carl Zeiss Inc., Oberkochen, Germany) scanning electron microscopes. Images were presented on a black background using Adobe Photoshop CS3 (Adobe Systems Incorporated, San Jose, CA, USA).

### DNA Extraction, PCR and Sequencing

Prior to DNA extraction, the tubes were centrifuged for 10 min at 10,000 r.p.m. to settle the cells of *Gambierdiscus* and *Goniodoma*, and the ethanol was aspirated. 100 μL of DNA lysis buffer (0.1 M EDTA pH 8.0, 1% SDS, 200 μg mL^−1^ proteinase K) was used to re-suspend the cell pellets and the resuspension was transferred into a 1.5 mL tube, and the process was repeated for five times. Then, the resultant 0.5 mL was incubated for 48 hours at 55°C. DNA extraction and purification followed a previously reported protocol [16]. At the end of the extraction process, DNA of *Gambierdiscus* or *Goniodoma* was eluted in 50 μl Tris-HCl solution. Next, 1 μL of the extracted DNA was PCR amplified. The SSU rDNA (∼1800 bp) of *Gambierdiscus* was amplified using the primers Dino18SF1 and 18ScomR1 [17]. The 3’end-SSU-ITS1-5.8S-ITS2-LSU (∼1900 bp) of *Gambierdiscus* was amplified using the primers Dino1662-F and 28S-R2 [17]. The SSU rDNA (∼1800 bp) of *Goniodoma* was amplified using the primers 18ScomF1 [18] and DinoR [19]. The 3’end-SSU-ITS1-5.8S-ITS2-LSU (∼1900 bp) of *Goniodoma* was amplified using the primers Gon-Lf (designed for this study; AATGAGTGTGTCATCTTGCC) and com28SR1 ([20], Table 1). The PCR amplifications were carried out in 25 μL reaction volumes containing 0.125 μL of TaKaRa Ex Taq HS in the PCR master mix (TaKaRa Bio, Dalian, China), both forward and reverse primers (final concentration 0.2 μM) and template DNA. Thermocycling conditions included a denaturing step of 94°C for 4 min; 35 cycles of 94°C for 30 s, 56°C for 30 sec, 72°C for 45 sec, and a final extension step of 72°C, 10 min. PCR products were resolved by agarose gel electrophoresis with the DL2000 DNA Ladder (Shanghai ShineGene Molecular Bio-tech Co) and the bands with expected sizes were excised in order to remove primer dimers. DNA was purified and directly sequenced as previously reported [16].

**Table 1:** Primers used in the present study.

Primer name	Application	Sequences (5'-3')	References
**Dino18SF1**	SSU for *Gambierdiscus*	AAGGGTTGTGTTYATTAGNTACARAAC	Qiu et al. [16]
**18ScomR1**	SSU for *Gambierdiscus*	CACCTACGGAAACCTTGTTACGAC	Qiu et al. [16]
**Dino1662 F**	LSU for *Gambierdiscus*	CCGATTGAGTGWTCCGGTGAATAA	Qiu et al. [17]
**28S R2**	LSU for *Gambierdiscus*	ATTCGGCAGGTGAGTTGTTAC	Qiu et al. [17]
**18ScomF1**	SSU for *Goniodoma*	GCTTGTCTCAAAGATTAAGCCATGC	Zhang et al. [18]
**DinoR**	SSU for *Goniodoma*	TTATTCACCGGAWCACTCAATCGG	Hoppenrath et al. [19]
**Gon-Lf**	LSU for *Goniodoma*	AATGAGTGTGTCATCTTGCC	This study
**com28SR1**	LSU for *Goniodoma*	TCACGCATAGTTCACCATCTTTCG	Wang et al. [20]

### Phylogeny and Sequence Analyses

SSU and SSU-ITS-LSU sequences of *Gambierdiscus* and *Goniodoma* were assembled from the Brazilian isolates obtained in this study. The new SSU rDNA sequences of *Gambierdiscus* and *Goniodoma* were aligned with sequences of *Gambierdiscus* spp., *Coolia* spp., *Ostreopsis* spp., *Alexandrium* spp., *Pyrodinium bahamense*, *Gonyaulax*, *Lingulodinium*, *Ceratocorys*, *Protoceratium*, *Ceratium*, *Neoceratium*, *Pyrocystis*, *Pyrophacus* and *Euduboscquella* available from GenBank database with *Oxyrrhis marina* (accession number #AF280077) as out-group in Mega 6.0.4. The new D1-D4 LSU rDNA sequence of *Gambierdiscus* and a new D1-D3 sequence of *Goniodoma* were aligned with sequences of *Gambierdiscus* spp., *Coolia* spp., *Ostreopsis* spp., *Alexandrium* spp., *Pyrodinium bahamense*, and *Goniodoma polyedricum* available from GenBank database with *Oxyrrhis marina* (accession number #EF613360) as out-group in Mega 6.0.4. Combined sequences were aligned using ClustalW using default parameters [21], and obvious misalignments adjusted manually. The SSU and trimmed D1-D3 LSU (1048 bp) alignments were then analyzed using ModelTest to select the most appropriate evolutionary model [22]. The selected General Time Reversible (GTR) model with gamma distribution was employed for Maximum Likelihood analysis using PhyML3.0 [23]. Categories of substitution rates were set at 4, and other parameters were estimated based on the datasets. The proportion of invariable sites and the gamma shape parameter were 0.252 and 0.574 for the SSU dataset, and 0.086 and 1.134 for LSU. The sequences obtained in this study were deposited in GenBank under accession numbers KM886379-KM886380.

### Nomenclature

The electronic version of this article in Portable Document Format (PDF) in a work with an ISSN or ISBN will represent a published work according to the International Code of Nomenclature for algae, fungi, and plants, and hence the new names contained in the electronic publication of a PLOS ONE article are effectively published under that Code from the electronic edition alone, so there is no longer any need to provide printed copies.

The online version of this work is archived and available from the following digital repositories: PubMed Central, LOCKSS.

## Results

Our data strongly support the split of the globular and lenticular species of *Gambierdiscus* into two distinct genera based on the cell shape, plate arrangement, and the considerable evolutionary distance of their respective SSU and LSU rDNA sequences, which form two well-separated clades. We propose a new genus name for a new species isolated from Brazil and the transfer of the other globular species of the genus *Gambierdiscus* into a new genus.

### Taxonomic Description

Alveolata Cavalier-Smith 1991; Dinophyceae G.S. West et Fritsch 1927; Gonyaulacales F.J.R. Taylor 1980; Goniodomataceae Er. Lindem. 1928

*Fukuyoa* gen. nov. F. Gómez, D. Qiu, R.M. Lopes & S. Lin, *sp*. *nov*.

#### Diagnosis

Cells are globular in shape, with a descending cingular displacement. Sulcus long, broad and not pouch-like morphology. Apical pore plate (Po) is centrally located in the epitheca with a long-shank fishhook-shaped slit. Plate formula Po, 3', 7'', 6c, 7s, 5''', 1p, and 2''''.

#### Type species

*Fukuyoa paulensis* F. Gómez, D. Qiu, R.M. Lopes & S. Lin, *sp*. *nov*. (see diagnosis below), hic designatus.

#### Etymology

In honor of Prof. Yasuwo Fukuyo, a pioneer in the study of epiphytic toxic dinoflagellates, and who first described the genus *Gambierdiscus*. The gender is feminine (ICN: Art. 60.11; Recom. 60C.1).

Other species belonging to *Fukuyoa*:

*Fukuyoa yasumotoi* (M.J. Holmes) F. Gómez, D. Qiu, R.M. Lopes & S. Lin, comb. nov.

**Fig 1:**
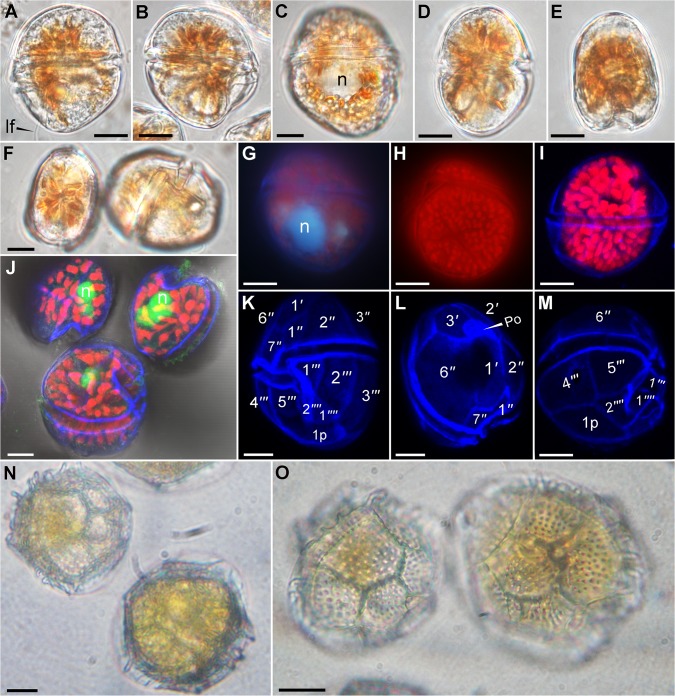
Light microscopy pictures of *Fukuyoa paulensis* gen. et sp. nov. (A-M) and *Goniodoma polyedricum* (N-O). (A-F) Live cells observed under a Zeiss Imager.A2 with Nomarski differential interference contrast (D.I.C.) optics at ×1000 magnification. (A) Left lateral view. Note the longitudinal flagellum. (B-C) Right lateral view. Note the nucleus. (D) Dorsal view. (E) Antapical view. Apical and ventral views. (G-H) Glutaraldehyde-fixed cells observed under an Olympus BX51 epifluorescence microscope at ×1000 magnification. (G) See the nucleus stained with DAPI. (H) Epifluorescence image of chloroplasts. (I-M) Glutaraldehyde-fixed cells observed under an inverted Leica TCS SP8 AOBS confocal microscope at ×630 magnification. The plates were stained with Fluorescent Brightener 28, and the nuclei were stained with SYBR Green I. (I) Overlay image of the cell showing the plastids. (J) See the nucleus stained with SYBR Green I. (K-M) Overlay image showing the thecal plates stained with Fluorescent Brightener 28. (N-O) Live cells of *Goniodoma polyedricum* isolated for PCR. Pictures taken at ×600 magnification with a Nikon TS-100 inverted microscope equipped with a SONY CyberShot DSC-W310 camera. n = nucleus; lf = longitudinal flagellum. Scale bar 10 μm.

Basionym: *Gambierdiscus yasumotoi* M.J. Holmes 1998 (J. Phycol. 34, 662, Fig. 1)

*Fukuyoa ruetzleri* (M.A. Faust, R.W. Litaker, M.W. Vandersea, S.R. Kibler, M.J. Holland & P.A. Tester) F. Gómez, D. Qiu, R.M. Lopes & S. Lin, comb. nov.

Basionym: *Gambierdiscus ruetzleri* M.A. Faust, R.W. Litaker, M.W. Vandersea, S.R. Kibler, M.J. Holland & P.A. Tester 2009 (Phycologia 48, 373, Fig. 43).

### *Fukuyoa paulensis* F. Gómez, D. Qiu, R.M. Lopes & S. Lin, *sp*. *nov*.

#### Diagnosis

Cells are globular in shape with average depth 50 ± 3 (45–56) μm, width 45 ± 2 (41–48) μm, and length 56 ± 3 (51–62) μm, and an average width-to-depth ratio of ∼1.2. Cells were broad in lateral view. Epitheca is dome-shaped and hypotheca slightly larger than epitheca. Thecal pores are round and numerous. Plate formula Po, 3', 7'', 6c, 7s, 5''', 1p, and 2''''. Apical pore plate is centrally located in the epitheca with a long-shank fishhook-shaped slit. First apical plate (1') is the biggest apical plate, broad and five-sided. Plate 2' is long, curved or straight in the suture 2'/3''. Plate 3' is broad and intermediate in size. Plate 1'' is minuscule and 7'' is also small, both lying in the wedge-shaped posterior end of plate 1'. Plates 3'' and 6'' are the biggest of the epitheca, and the 2'', 4'' and 5'' are intermediate in size. The descending cingulum was displaced twice its width. The sulcus is deep and excavated, with two prominent posterior sulcal plates. Plates 1''' and 5''' are small, plate 3''' median in size, and plates 2''' and 4''' are the largest of the hypotheca. The rectangular plate 1'''' is small, and folded onto the left side of the sulcus. Plate 2'''' is forked and invades the base of the sulcal hollow. There is one intercalary posterior plate (1p), with the shape of an elongated pentagon situated between plates 2''' and 4'''. Nucleus situated in the hypotheca. The cells contain numerous small ellipsoidal plastids with a brownish pigmentation (Fig. 1).

#### Holotype

**Fig 2:**
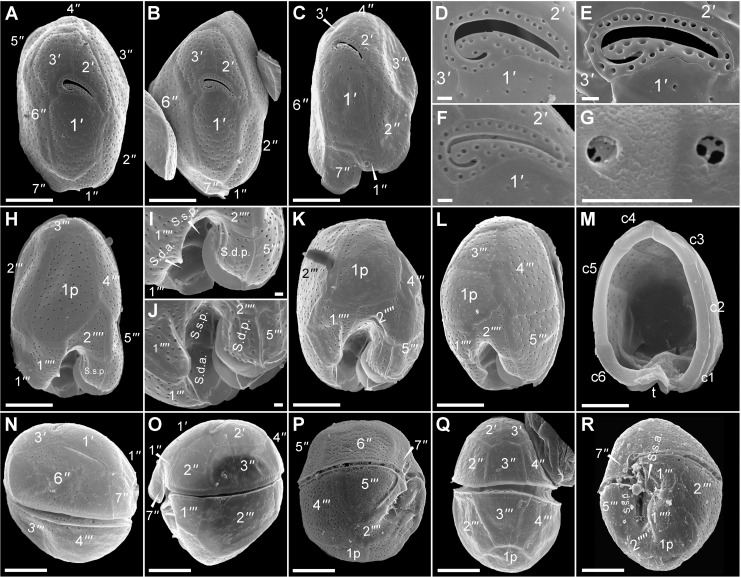
SEM micrographs of *Fukuyoa paulensis* gen. et sp. nov. (A-C) Apical views. (D-F) Apical pore plate, Po. (G) Detail of the pores. (H-L) Antapical views. (I-J) Detail of the sulcal plates in antapical view. (M) Cingular plates. (N) Right lateral view. (O) Left lateral view. (P) Right ventro-lateral view. (Q) Dorsal view. (R). Ventral view. Scale bar 10 μm, except D-G, I-J, scale bar 1 μm.

Fig. 2A collected by F. Gómez. SEM stub #410, Type Collection of Dinoflagellates, National Museum of Natural History, Smithsonian Institution. The culture strain is barcoded in GenBank by the following SSU 1–1699, D1–D4 LSU 2139–3450 rDNA, and ITS 1700–2138 sequences (#KM886379). Isolate obtained as epiphyte on macroalgae at <1 m depth during the low tide at Ubatuba, Brazil.

#### Isotypes

Fig. 2B-R; SEM stub #410, Type Collection of Dinoflagellates, National Museum of Natural History, Smithsonian Institution.

#### Synonyms

*Gambierdiscus* cf. *yasumotoi* sensu Rhodes et al. [31], *Gambierdiscus yasumotoi* sensu Murray et al. [32].

#### Etymology

*paulensis* (Latin) refers to the geographic locality, the coasts of São Paulo State, where the species was isolated.

#### Type locality

Saco da Ribeira, Ubatuba, São Paulo State, Brazil (23° 30' 3.09" S, 45° 7' 7.32" W).

#### Distribution

It is found as epiphyte in macroalgae and sands around the pier of the Marine Station of the University of São Paulo at Ubatuba. It is often associated with proliferations of *Prorocentrum rhathymum* that usually occurred in shallow waters during high light and warm periods. In addition to the observations of wild cells in benthic habitats, specimens were found in surface plankton samples collected at São Sebastião Channel (23° 50' 4.05'' S, 45° 24' 28.82'' W, water column depth of 40 m), and also offshore Ubatuba (23° 32' 20.15" S, 45° 5' 58.94" W, water column depth of 30 m). The D1–D3 LSU rDNA sequences AB859986 and KM272973 retrieved from GenBank as *Gambierdiscus* cf. *yasumotoi* strain CAWD210 isolated from New Zealand and *Gambierdiscus yasumotoi* strain NQAIF210 isolated from Australia, respectively, are identical to that of *Fukuyoa paulensis*.

### Morphology

**Fig 3:**
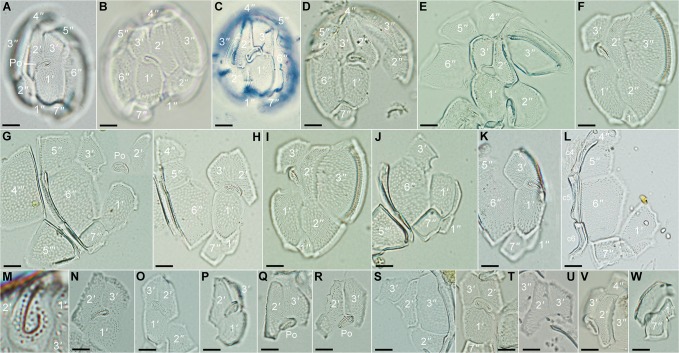
Light micrographs of dissected epithecal plates of *Fukuyoa paulensis* gen. et sp. nov. (A-F) Epithecal plates. (G-L) Precingular and apical plates. (M) Apical pore plate. (N-V) Apical plates. (W) Precingular plates 1'' and 7''. Scale bar 10 μm.

Armored globular cells, with average depth (dorso-ventral axis) 50±3 (45–56) μm, width 45±2 (41–48) μm, and length 56±3 (51–62) μm, with an average depth-to-width ratio of ∼1.2 (Fig. 1A-F). The cells possess small ellipsoidal plastids, 3–4 μm in diameter, with a brownish pigmentation (Fig. 1A-F, H-J). The nucleus (15–20 μm in diameter) is globular and situated in the hypotheca (Fig. 1C,G,J). The height of the hypotheca is slightly higher than the epitheca (Fig. 1A-D). In apical or antapical view the cell is oval and indented in the ventral side (Fig. 1E-F). The whole theca is covered with round pores of about 0.35 μm in diameter and at densities of 40–50 per 100 μm^−2^ (Fig. 2G,N-Q). The pores are evenly distributed. Plate formula is Po, 3', 7'', 6c, 7s, 5''', 1p and 2''''. Apical pore plate is an elongate ellipsoid, of 10–12 μm long and 6–7 μm wide, and centrally located in the epitheca (Fig. 2A-C, 3A-E). Po plate has a long-shank fishhook-shaped slit, about 8 μm long, surrounded by a row of marginal pores, and a few dispersed internal pores (located inside the curved extreme of the foramen) (Fig. 2D-F, 3M). Apical pore plate contacts three plates: 1', 2' and 3' (Fig. 1L, 2A-F). Apical plate 1' is the biggest of the apical series, 20–28 μm long and 17–18 μm wide, with a pentagonal shape (Fig. 3A,B,D,K). Plate 1' is posteriorly wedge-shaped in the junction with the 1'' and 7'' plates (Fig. 2A-C, 3G-L). In the dorsal side of the epitheca, the sum of plates 2' and 3' is 20–23 μm wide, while in the ventral side plate 1' is 17–18 μm wide. Plate 2' is elongate rectangular (30–33 μm long), and narrower (10–12 μm) than the other apical plates. It has a short suture with plates 1', and 2'' (Fig. 3A-F). The suture 2'/3'' is long, straight (Fig. 3B,N,Q-S) or curved (Fig. 3I,V). The suture between the plates 2'/3'' is sometimes dentate (Fig. 3R-S). The shape of plate 2' resembles an angel’s wing (Fig. 3S). Plate 3' is the smaller apical series (about 10–13 μm wide), with an irregular five-sided contour, and with the apical pore plate occupying one of the vertices. The suture 2'/3' is about 20 μm long, while the suture 3'/4' is short (Fig. 2A-C, 3D-E).

Plate 1'' is four-sided and the smallest of the precingular series, followed by plate 7'' (Fig. 2A, 3H,J-K). Both plates are lying adjacent to the sulcus and in the posterior end of the wedge- plate 1' (Fig. 2A-B, 3A-D,H). Plates 3'' and 6'' are the biggest of the epitheca (Fig. 2O-Q, 3B,E,F). Plate 6'' is pentagonal and the suture 1'/6'' is about twice as long as the suture 3'/6'' (Fig. 2N,P, 3G-H). The suture 2'/3' is straight (Fig. 3S) or slightly curved (Fig. 3I). Plates 4'' and 5'' are four-sided, trapezium-shaped, and intermediate in size (about one half the area of plates 3'' or 6'') (Fig. 3D,E). Plate 2'' is slightly bigger than plates 4'' or 5'' (Fig. 3E).

**Fig 4:**
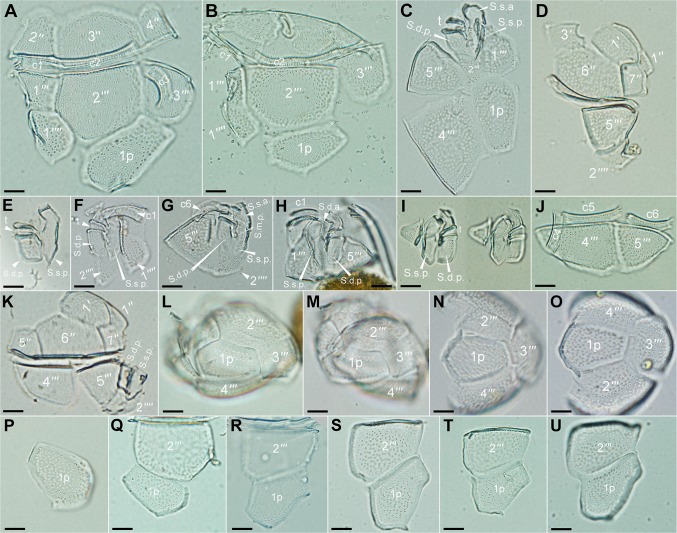
Light micrographs of dissected hypothecal, sulcal and cingular plates of *Fukuyoa paulensis* gen. et sp. nov. (A-D) Hypothecal plates. (E-I) Sulcal plates. The tiny sulcal median anterior (S.m.a.) plate that was not discernible. (J-K) Postcingular and cingular plates. (L-P) Antapical view of the cells. (P-U) Detail of the first posterior intercalary plate (1p). Scale bar 10 μm.

The cingulum is descending and displaced twice its width (Fig. 1F). The cingulum is bordered by a narrow list and contains six narrow plates about 4 μm wide (Fig. 3L, 4A-B,J). The sulcus is deep and excavated, mostly surrounded by a ridged sulcal list (Fig. 2H-L,P,R). The sulcus consists of seven plates, namely S.d.a., S.d.p. [sulcal (dexter = right) anterior or posterior], S.s.a., S.s.p. [sulcal sinister (= left) anterior or posterior], t (transitional cingular plate), S.m.a. and S.m.p. (sulcal media anterior or posterior) (Fig. 4E-I,K). The two posterior sulcal plates (s.d.p, s.s.p) are lying in the base of the sulcal hollow and anteriorly placed adjacent to the forked plate 2'' (Fig. 4E-I,K). The S.m.a. and S.m.p. plates are minuscule. The S.m.a. resides between the S.d.a. and transitional cingular (t) plates, whereas the S.m.p. is located below the t plate and adjacent to the S.d.p. plate (Fig. 3G). Plate t lies parallel to the upper surface of plate S.d.p. (Fig. 2M, 4E-G,I).

**Fig 5:**
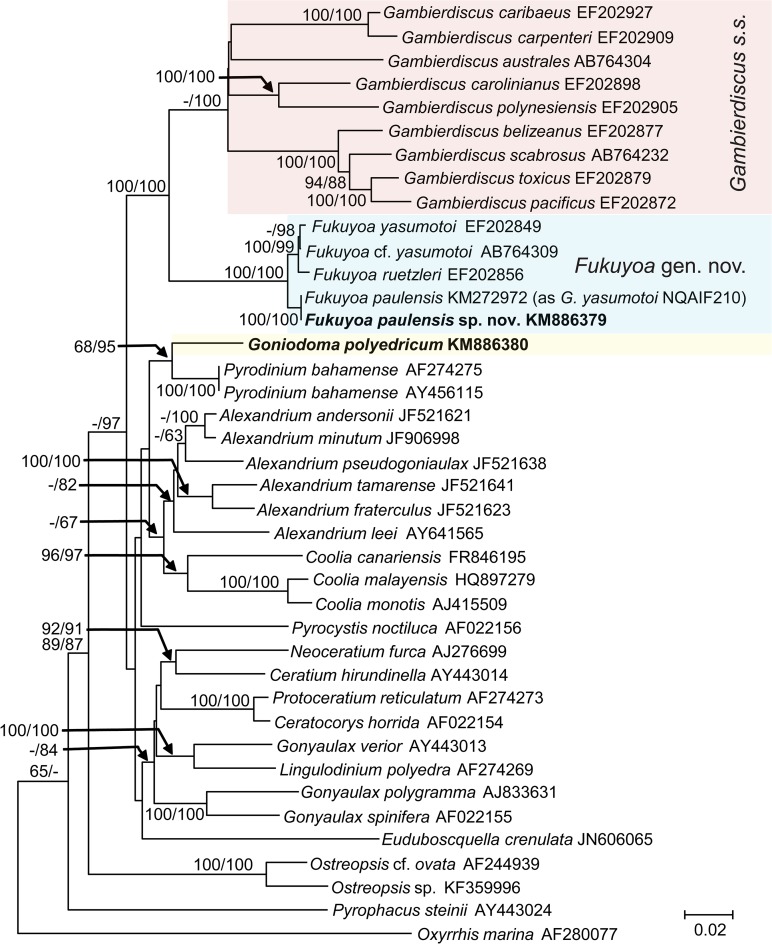
SSU rDNA-based phylogeny of *Fukuyoa paulensis* gen. et sp. nov. and *Goniodoma polyedricum* with some gonyaulacoid dinoflagellates. Sequences obtained in this study are bold-typed. Support of nodes is based on bootstrap values of ML/NJ with 1000 and 500 resamplings, respectively. Only values greater than 60 are shown. *Oxyrrhis marina* was used as outgroup.

The hypotheca also contains five postcingular plates, one large intercalary posterior plate (1p) and two small antapical plates (Fig. 2H-L, 4K-U). Plate 1''' is trapezoidal and the smallest of the postcingular plates (Fig. 2H-L,O). Plates 2''' and the 4''' are the biggest of the postcingular series (Fig. 2O,P,4O). Plate 2''' is five-sided (Fig. 4O,P-U) and the suture 2'''/1p is three times longer than in the suture with plate 1'''' (Fig. 2H, 4A-B). Plate 4''' is quadrangular (Fig. 4C). Plate 3''' and 5''' are intermediate in size (Fig. 2P,Q). Plate 3''' is trapezoidal (Fig. 4A,B) and 5''' is triangular (Fig. 4D,K). Plate 1p is long, broad and pentagonal in shape (Fig. 2H,K-L, 4O-U). It usually remains attached to plate 2''' after the theca dissection (Fig. 4A-B, Q-U). The size of the 1p plate is between 33–39 μm long, and 19–23 μm wide. The suture length is small with plate 3''' (of about 10–12 μm) and large with plate 2'' (Fig. 2Q, 4L-O). The antapical plates 1'''' and 2'''' are intermediate in size. Plate 1'''' is more or less quadrangular in shape (Fig. 2M, 4A,B), and lies immediately posterior to plate 1''' and adjacent to the sulcus, 2''' and 1p. Plate 2'''' is small (about 19 μm long, 10 μm wide) and placed posteriorly to plate 5''' (Fig. 2H-L, 4C). It invades the sulcus with a forked side in contact with the right and left posterior sulcal plates (Fig. 2I-K,P,R, 4K).

### Toxicity

The toxicity was tested from cell pellets using a mouse neuroblastoma assay according to the procedure described in Xu et al. [24] (see method as S1 File). The results did not reveal the presence of ciguatoxins.

### Molecular phylogeny

We obtained the sequence of a long rDNA fragment from the culture population of *Fukuyoa paulensis* (3450 base pairs, bp). This sequence contained SSU, ITS1-5.8S-ITS2, and partial LSU (D1-D4). For *Fukuyoa*, the SSU region that we obtained spanned 1699 bp and the LSU region 1312 bp. BLAST analyses showed that the SSU rDNA sequence of *F*. *paulensis* was identical to *G*. *yasumotoi* NQAIF210 (KM272972) from Australia and most similar to the *G*. cf. *yasumotoi* IR4G (AB764309) from Japan (99% identity, differing by 22 bp in 1699), followed by other sequences of *Fukuyoa yasumotoi* and *F*. *ruetzleri*. In this study we have obtained the first SSU rDNA sequence of *Goniodoma polyedricum*, which showed higher similarity to that of *F*. *paulensis* (87% identity, differing by 189 bp in 1534) than to that of other *Fukuyoa* species. The Fig. 1N-O illustrates the specimens used for PCR analysis. The sequence of the type of *Gambierdiscus*, *G*. *toxicus* GTT-91 (EF202878) and *F*. *paulensis* differed by 287 bp in 1681 (83% identity).

The LSU rDNA sequence of *F*. *paulensis* was identical to that of *G*. cf. *yasumotoi* strain CAWD210 (AB859986) from New Zealand and *G*. *yasumotoi* NQAIF210 (KM272973) from Australia, recently available at GenBank. *Fukuyoa paulensis* was closely related (96% identity, differing by 37 bp out of 969 bp) to that of *F*. *ruetzleri* (EF202962, EF202964) and *F*. *yasumotoi* (EF202967, EF202966), and closely related (95%, differing by 46 bp out of 969 bp) to the sequence of *F*. cf. *yasumotoi* (AB548852). A LSU rDNA sequence of *Goniodoma polyedricum* was available in GenBank (JQ247712). The sequence of *G*. *polyedricum* (JQ247712) was the closer to that of *F*. *paulensis* (74% identity, differing by 346 bp out of 1313 bp) than to sequences of other *Fukuyoa* spp. The LSU rDNA sequence of the type of *Gambierdiscus*, *G*. *toxicus* GTT91 (EF202951), and *F*. *paulensis* differed by 360 bp in 970 (63% identity).

**Fig 6:**
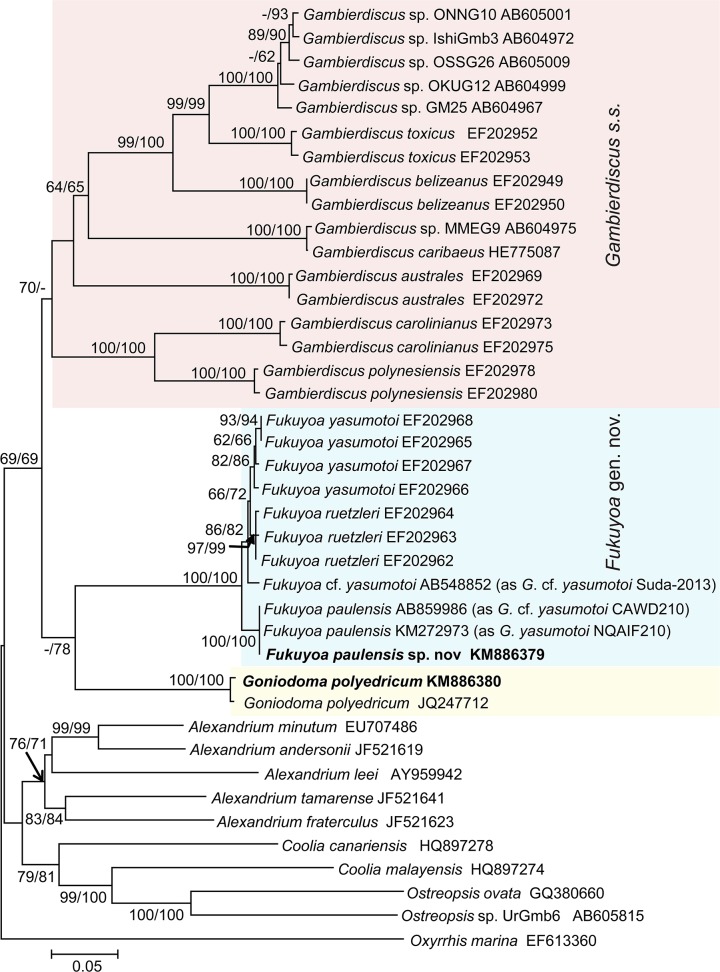
(D1-D3) LSU rDNA-based phylogeny of *Fukuyoa paulensis* gen. et sp. nov. and *Goniodoma polyedricum* with some gonyaulacoid dinoflagellates. Sequences obtained in this study are bold-typed. Support of nodes is based on bootstrap values of ML/NJ with 1000 and 500 resamplings, respectively. Only values greater than 60 are shown. *Oxyrrhis marina* was used as outgroup.

Phylogenetic trees based on SSU and LSU regions of rDNA were inferred separately, including 40 sequences respectively, representing all known clades of *Gambierdiscus sensu lato* and including some other gonyaulacoid lineages. For each sequence region, the topologies for Neighbor Joining (NJ) and Maximum Likelihood (ML) trees were similar, consistently placing *Fukuyoa paulensis* in sister relationship with *F*. *yasumotoi* and *F*. *ruetzleri* with moderate (SSU rDNA) to strong (LSU rDNA) NJ and ML supports. This supports the consideration as an independent species for *F*. *paulensis*. Meanwhile, in all these trees, the monophyltetic clade of *Fukuyoa* spp. was clearly separated from *Gambierdiscus s*.*s*. (= *sensu stricto*) (Figs. 5 and 6). In the SSU rDNA phylogeny, the subclade of *Fukuyoa* spp. branched between *Gambierdiscus s*.*s*. and *Goniodoma* (Fig. 5). In the LSU rDNA phylogeny, the species of *Fukuyoa* and *Goniodoma polyedricum* formed a weakly supported subclade that branched as a sister group of *Gambierdicus s*.*s*. (Fig. 6). These results support the consideration of an independent genus for the species of *Fukuyoa*.

## Discussion

### Split of *Gambierdiscus* sensu lato into *Fukuyoa* and *Gambierdiscus* s.s.

The SSU and LSU rDNA phylogenies indicate that the globular species, *Fukuyoa paulensis*, *F*. *ruetzleri* and *F*. *yasumotoi* are separated from the anterioposteriorly compressed morphology that characterizes *Gambierdiscus s*.*s*. (Fig. 5–6). The separation has very strong bootstrap support. Besides the contrast in general shape, *Fukuyoa* and *Gambierdiscus s*.*s*. also differ in plate morphology and pattern. *Fukuyoa* apical series has plate 1' is the largest, while in *Gambierdiscus s*.*s*. the largest plate is 2'. The sulcus of *Fukuyoa* shows seven plates rather than six plates; and plate 2'''' invades the sulcus in the globular species as occurs in other genera such as *Alexandrium* and *Goniodoma* [25,26].

In previous molecular phylogenies, *F*. *yasumotoi* and *F*. *ruetzleri* branched in a basal position to *Gambierdiscus s*.*s*. Litaker et al. [11] hypothesized these globular species as evolutionary intermediates in the transitional phase between the more ancestral globular morphology and the lenticular shapes of *Gambierdiscus s*.*s*. With no doubt, the genera *Fukuyoa*, *Gambierdiscus* and *Goniodoma* share a common ancestor (Figs. 5 and 6).

**Fig 7:**
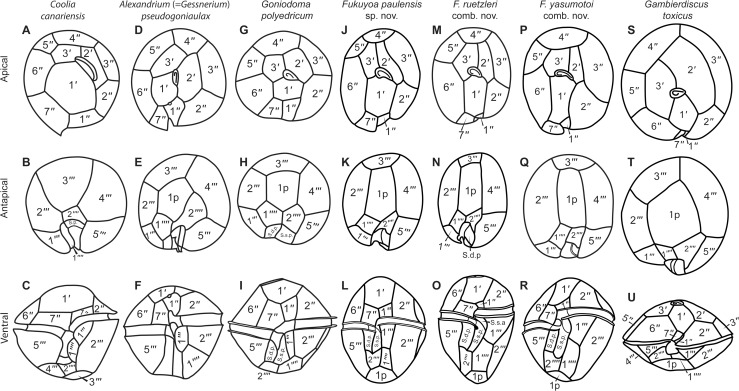
Line drawings of members of the family Goniodomataceae in apical, antapical and ventral views. (A-C) *Coolia canariensis* redrawn from Fraga et al. [27]. (D-F). *Alexandrium pseudogoniaulax* redrawn from Balech [25]. (G-I) *Goniodoma polyedricum* redrawn from Balech [25]. (J-L) *Fukuyoa paulensis* gen. et sp. nov. (M-O) *F*. *ruetzleri* comb. nov. redrawn from Litaker et al. [11]. (P-R) *F*. *yasumotoi* comb. nov. redrawn from Litaker et al. [11]. (S-U) *G*. *toxicus* redrawn from Litaker et al. [11].

Fig. 7 and Table 2 compare the apical, antapical and ventral views of the three globular species, and type species of *Gambierdiscus*, *G*. *toxicus*, representative of the more derived anterioposteriorly compressed forms, and the goniodomatacean genera *Goniodoma*, *Alexandrium* and *Coolia*. The migration from the planktonic to the benthic habitat of the ancestor of *Gambierdiscus s*.*s*. is associated with the development of anterioposteriorly compressed cell body with lenticular shape. The evolution in *Gambierdiscus* seems to be associated with the increase of cell size, high anterior-posterior compression with a lenticular cell shape in ventral view, ascending cingular displacement with pouch-like sulcal morphology (Table 2, Fig. 7). *Goniodoma* possesses a rotund shape in apical or antapical views, while the cells of *Fukuyoa* are more ellipsoidal, mainly associated with an elongation of the apical plates. The lenticular species, *Gambierdiscus s*.*s*., exhibit a proportional reduction of the first apical plate (1'), and the precingular plates 7'' and 1'' in the epitheca. There is a size increase of the first posterior intercalary (1p) plate in the hypotheca (Fig. 7). Some lenticular species tend to have a broad plate 1p (*G*. *caribaeus*, *G*. *carolinianus*, *G*. *carpenteri*), when compared to the narrower and quadrangular shape of *Fukuyoa* spp. Plate 1' is almost hexagonal in *Gambierdiscus s*.*s*., nearly rectangular in *F*. *yasumotoi*, and narrow pentagonal in *F*. *ruetzleri*. In contrast, *F*. *paulensis* has a broad pentagonal plate 1' that is closer to the more regular pentagonal shape of plate 1' in *Goniodoma* (Fig. 7). Plate 2' is broad rectangular in *Gambierdiscus s*.*s*. and narrow rectangular in *Fukuyoa*. Plate 3' is variable in shape, usually with round border in *Gambierdiscus s*.*s*., while it is pentagonal with marked border corners in *Fukuyoa*. The sulcus of *Fukuyoa* is longer and broader than in *Gambierdiscus s*.*s*. (Fig. 7).

**Table 2:** Comparison of morphological characteristics of genera of the family Goniodomataceae.

	*Coolia*	*Alexandrium* (= *Gessnerium*)	*Goniodoma*	*Fukuyoa* gen. nov.	*Gambierdiscus s*.*s*.
***Shape in ventral view***	globular	globular	globular	globular	lenticular
***Shape in apical view***	round	round	round	elliptical	round
***Cell compression***	slight anterior-posterior	not compressed	not compressed	slight lateral	High anterior-posterior
***Po position***	dorsal	central	central	central	central
***Po shape***	short, curved slit	short, curved slit	short-shank fishhook	long-shank fishhook	short-shank fishhook
***Largest apical***	1′	similar apicals	similar apicals	1′	2′
***Shape 1′***	oblong, hexagonal	pentagonal	pentagonal	oblong, hexagonal	rhomboidal
***Shape 2′***	long hexagonal	wide pentagonal	wide rectangular	long narrow	wide hatchet shaped
***Cingular displacement***	descending	descending	descending	descending	ascending
***Sulcal morphology***	not pouch-like	not pouch-like	not pouch-like	not pouch-like	pouch-like
***Plate 1p***	no	wide	wide	narrow	wide
***Antapical plates***	2′′′′> 1′′′′	2′′′′1′′′′	2′′′′∼ 1′′′′	2′′′′∼ 1′′′′	2′′′′> 1′′′′

There are discrepancies in the nomenclature of the thecal plates, especially in the apical series. Litaker et al. [11] described four new species of *Gambierdiscus s*.*s*. and one species now classified as *Fukuyoa* with the tabulation Po, 3', 7'', 5''', 1p and 2'''', while later Fraga et al. [6] reported plate formula as Po, 4', 6'', 5''', 0p, 2'''' for a new species of *Gambierdiscus s*.*s*. Fraga et al. used the modified Kofoid tabulation system as described in Besada et al. [28]. The differences are whether to consider the first apical plate (1') as first precingular plate (1'') and in the hypotheca, whether the second antapical plate (2'''') is considered as 1p, and sulcal posterior (S.p.) as second antapical. The small plate 1'' (plate 1' in Fraga et al. [6]) does not contact with the apical pore plate. If we consider the close phylogenetic relationship between *Goniodoma* and *Fukuyoa*, we can consider the same epitheca tabulation described for *Goniodoma*. Balech [25] provided the epithecal formula Po, 3', 7'' for *Goniodoma* that have three quite similar apical plates, and there is no reason to consider that one of the precingular plates belongs to the apical series.

In addition, there are discrepancies in the tabulation of the hypothecal plates. There are two plates, 1''' and 2'''', in contact with the sulcus. Plate 2'''' of *Fukuyoa* invades the sulcus, and the two sulcal posterior plates are placed in its forked side. Plate 2'''' also invades the sulcus in species of *Alexandrium* or *Goniodoma*, but not in *Gambierdiscus s*.*s*. [11]. When the plate to the right of plate 1'''' invades the sulcus, as in *Fukuyoa*, it can be logically assigned to the sulcal series (S.p.) and the remaining antapical-most plate designated as plate 2'''' as in the related *Alexandrium* species [11,25]. This interpretation takes into consideration that plate 2'''' is a sulcal posterior plate, and plate 1p is not touching the cingulum (tabulation 0p, 2''''). Balech [29] used the term perisulcal (= antapical) for plates in contact with the sulcus. Plate 1p of *Gambierdiscus s*.*s*. is not adjacent to the sulcus, and consequently the other two plates, 1'''' and 2'''', are considered as antapicals. Plate 2'''' of *F*. *paulensis* is forked, and two sulcal plates, the S.d.p. and S.s.p., are fixed on the fork-shaped plate 2''''. We have not found a compelling reason to consider plate 2'''' as a sulcal plate even if it invades the sulcus.

### Differences among species in *Fukuyoa*

In dorso-ventral view, the contour of *F*. *ruetzleri* (= *G*. *ruetzleri*) is narrow, while *Fukuyoa paulensis* is more rotund and more similar to *F*. *yasumotoi* (= *G*. *yasumotoi*). *Fukuyoa paulensis* is intermediate in size between *F*. *yasumotoi* and *F*. *ruetzleri*. According to Litaker et al. [11] the cell dimension of *F*. *yasumotoi* is depth 57 ± 5 (49–67) μm, width 52 ± 5 (43–60) μm and length 62 ± 4 (54–68) μm and for *F*. *ruetzleri* is depth 45 ± 3 μm (range 42–55 μm), width 37 ± 3 (31–42) μm, length 52 ± 5 (45–59) μm. The dimension of our new species is depth 50 ± 3 (45–56) μm, width 45 ± 2 (41–48) μm, and length 56 ± 3 (51–62) μm. Litaker et al. proposed a dichotomous tree for *Gambierdiscus*, where globular cells with <42 μm cell width corresponded to *F*. *ruetzleri*. Based on the size, the smaller specimens of *F*. *paulensis* fall into the size range of *F*. *ruetzleri*, and most of the specimens are closer to *F*. *yasumotoi*. However, the overlapping in the size range between the species suggests the need to use other morphological character as diagnostic character.

Plate 1' of *F*. *paulensis* is broader and occupies a proportionately larger portion of the epitheca than in the other species. Plate 1' is nearly rectangular in *G*. *yasumotoi* or narrow pentagonal in *F*. *ruetzleri*, while broad pentagonal in *F*. *paulensis*. Consequently, the main diagnostic morphological character is that *F*. *paulensis* has a broad plate 1' in comparison to the narrow plate 1' of the other globular species (Fig. 7). Plate 2' is more elongated and occupies a larger area with respect to plate 3' than in other globular species. Plate 3' is more reduced in *F*. *paulensis* and the five-sided contour is less apparent because the suture with plate 4''' is shorter. Posterior intercalary plate (1p) is an elongated pentagon in *F*. *paulensis*, broader than in the other globular species (Fig. 7). Consequently, the tabulation (mainly the apical series) is the main diagnostic morphological character.

Besides morphological distinction, our phylogenetic inference also showed separation of the three species. In particular, branching of *F*. *paulensis* from the other two was clear and very strongly supported. Despite the short branch lengths in general, there is still notable difference between the interspecific variation and intraspecific variation. The geographic populations of each species, particularly *F*. *paulensis* and *F*. *ruetzleri*, showed largely identical sequences of the 18S and 28S rDNA fragments. The only exception is the newly reported *F*. cf. *yasumotoi* from Japan (AB548852) that appeared to be an intermediate between *F*. *paulensis* and the other two species. Until recently *F*. *yasumotoi* and *F*. *ruetzleri* had not been found outside of the tropical Pacific and North Atlantic, respectively [3, 30]. New strains have now been reported from Japan [15], New Zealand [31], and Australia [32]. The D1-D3 LSU rDNA sequences of the strains isolated from New Zealand and Australia, and the SSU rDNA sequence from the Australian strain were identical to *Fukuyoa paulensis*, and these sequences from the southern hemisphere forms a distinctive subclade. Further studies with broader sampling of different geographic strains are required to more precisely resolve all these taxa. Numerous regions of the world´s oceans remain under-investigated and our knowledge of the biogeography and distribution of *Gambierdiscus sensu lato* remains incomplete.

## Supporting Information

S1 File Toxicity test method. Description of mouse neuroblastoma assay (MNA) for ciguatoxins.
(PDF)
