# Supplementary material for: Fukuyoa paulensis gen. et sp. nov., a New Genus for the Globular Species of the Dinoflagellate Gambierdiscus (Dinophyceae)
Source: PLoS One. 2015 Apr 1;10(4):e0119676. doi: 10.1371/journal.pone.0119676 (PMC4382304; doi:10.1371/journal.pone.0119676)
Supplement: S1 File — Description of mouse neuroblastoma assay (MNA) for ciguatoxins. (PDF) [file pone.0119676.s001.pdf]

Toxicity test method.

Description of mouse neuroblastoma assay (MNA) for ciguatoxins.

Yim Ling Mak & Leo Lai Chan

State Key Laboratory in Marine Pollution, Research Centre for the Oceans and Human Health, City University of Hong Kong, Hong Kong Special Administrative Region

Extraction

Ciguatoxins (CTXs) were extracted from *Gambierdiscus* cell pellets according to the procedures described in Xu et al. (2014). Briefly, cell pellet was extracted in methanol under sonication for 30 minutes. After centrifugation at 4000 rpm for 15 minutes, supernatant was collected. The extraction was repeated twice and all the supernatant was combined. After the extract was evaporated, a solvent partition was applied to the resulting residue using dichloromethane and 60% aqueous methanol for three times. The dichloromethane soluble fractions (DSFs), in which CTXs were recovered, were dried under vacuum and stored at –20°C until tested for toxicity via mouse neuroblastoma assay (MNA).

Mouse neuroblastoma assay (MNA)

Mouse neuroblastoma (Neuro-2a cells) (ATCC, CCL131; ATCC, Manassas, VA) were cultured in Roswell Park Memorial Institute (RPMI)-1640 medium (Gibco, Life Technologies, Carlsbad, CA) that was supplemented with 10% fetal bovine serum (HyClone, Thermo Fisher Scientific, Waltham, MA), 2 g/L Na_2CO_3, antibiotic solution (50 units/mL penicillin and 50 µg/mL streptomycin) and 2.5 µg/mL Fungizone® (Gibco Life Technologies, Carlsbad, CA) at 37°C in 5% CO_2. Cells were seeded at a density of 2.5 x 10^5 cells/mL in 96-well plate. After 24-hour incubation, medium was renewed with complete RPMI-1640 containing 0.1 mM ouabain and 0.01 mM veratridine. Cells were dosed with 10 uL per well extracts in three replicates. After 18-hour incubation, cell viability was measured by MTT [3-(4, 5-dimethyl-thiazol-2-yl)-2, 5-diphenyltetrazolium bromide] assay. Absorbance was measured using a microplate reader (Molecular Devices Spectra Max 340 PC) at 595 nm with a reference wavelength of 655 nm.

The optical density acquired for each well was normalized by the MTT blank. Toxicities of *Gambierdiscus* spp. were determined from the standard curve.

Cells were dosed with 10 µL/well P-CTX-1 standards at seven concentrations ranging from 9.77 pg/mL to 313 pg/mL in five replicates. A standard curve of P-CTX-1 was plotted using non-linear regression ($r^2 < 0.990$). All the toxicity value of fish samples were determined based on the standard curve with a limit of quantification (LOQ) ranged from 9.77×10^{-8} to 9.77×10^{-7} pg P-CTX-1 eq /cell. Quality control of the assays was performed by testing each MNA with P-CTX-1 standard of 39.1 pg/mL. The assays were conducted twice and the toxicity values are reported as mean P-CTX-1 eq between two assays. The inter-plate relative standard deviation was 4.84%, and inter-assay relative standard deviation was 4.85%.

Reference

Xu Y, Richlen ML, Morton SL, Mak YL, Chan Lai L, Tekiau A, Anderson DM (2014) Distribution, abundance and diversity of *Gambierdiscus* spp. from a ciguatera-endemic area in Marakei, Republic of Kiribati. Harmful Algae 34: 56-68.
